# Pinwheel-Shaped Titanium Plates Should Be Fixed to the Skull Using All Screw Holes to Protect the Plates from Being Bent

**DOI:** 10.1155/2019/5709285

**Published:** 2019-08-19

**Authors:** Shoko Merrit Yamada, Katsuya Gorai, Koichi Gonda

**Affiliations:** ^1^Department of Neurosurgery, Teikyo University Mizonokuchi Hospital, 5-1-1 Futago, Takatsu-ku, Kawasaki, Kanagawa 213-8507, Japan; ^2^Department of Plastic Surgery, Teikyo University Mizonokuchi Hospital, 5-1-1 Futago, Takatsu-ku, Kawasaki, Kanagawa 213-8507, Japan

## Abstract

**Introduction:**

In cranioplasty, pinwheel-shaped titanium mini plates are frequently used to cover bone defects produced by burr holes, and it is common to insert screws through only a few of the holes in cranial flap fixation.

**Presentation of Case:**

A 69-year-old man who had undergone clipping surgery for subarachnoid hemorrhage 16 years previously visited our clinic because a titanium plate had penetrated his scalp one month after he was hit on the head by a wall cabinet. Imaging studies revealed that part of the titanium plate had bent outwards and penetrated the skin. The plate was surgically removed, a relief skin incision was made 6 cm posterior to the skin defect to suture the defected portion without causing tension, and a skin graft was applied to the relief skin incision portion. Two months after the maneuver, the skin graft had been successfully incorporated without infection.

**Discussion:**

Even after the subcutaneous and the cutaneous tissue have completely covered the pinwheel-shaped titanium mini plate, an edge without screw fixation can be easily bent by a hard blow to the overlying scalp. We recommend fixation of pinwheel-shaped titanium plates used in cranioplasty through all screw holes to protect against the plate being bent.

## 1. Introduction

Titanium mini plates are indispensable in cranioplasty, contributing to a good cosmetic result [1–5]. Pinwheel-shaped titanium mini plates are frequently used to cover burr-hole defects. Such plates have several holes for screw fixation, but not all holes are used because four screws are enough to achieve stable bone flap fixation. We report here a case of plate protrusion at an unfixed edge of a pinwheel-shaped titanium mini plate after the patient was hit on the head.

## 2. Case Presentation

**Figure 1:**
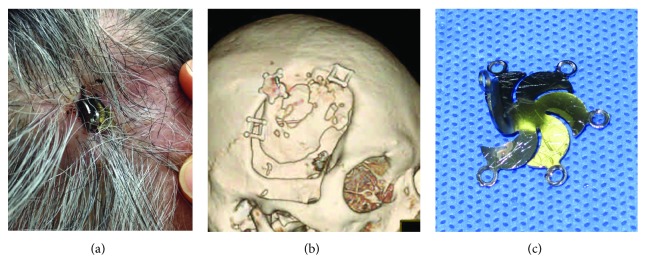
Change in shape of a pinwheel-shaped titanium mini plate. (a) A titanium plate that had been inserted during cranioplasty 16 years previously is protruding from the scalp through a circular defect. (b) Three-dimensional computed tomography (3D-CT) scan image showing that part of the titanium plate has been bent upwards. (c) After surgical removal, the bend in the plate is clearly visible.

A 69-year-old Asian man noticed a titanium plate penetrating through his scalp in the right temporal region one week prior to visiting our clinic. The patient had a history of subarachnoid hemorrhage and had undergone clipping surgery of a ruptured middle cerebral artery aneurysm with pterional craniotomy 16 years previously. One month prior to visiting our clinic, he hit his right temporal region on a wall cabinet and noticed something hard bulging subcutaneously at the point of impact. Three weeks after the head trauma, he noticed the plate protruding through his scalp (Figure 1(a)). Three-dimensional computed tomography (3D-CT) scan revealed that part of the titanium plate had been bent and was penetrating the skin (Figure 1(b)). The plate was removed and the skin defect repaired.  Figure 1(c) shows the removed plate and the bend in it.

**Figure 2:**
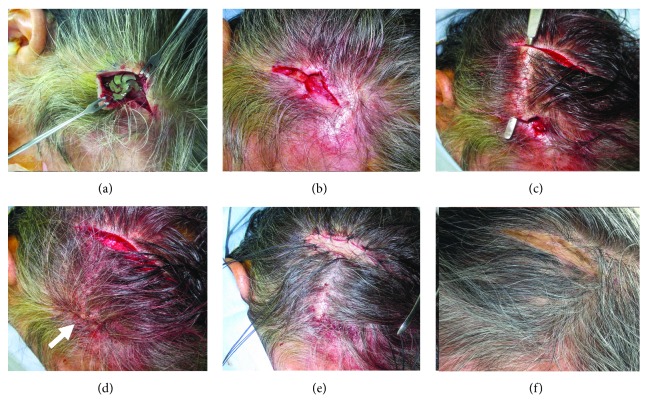
Surgical treatment. (a) A skin incision has been made above the titanium plate to expose it. (b) The plate and all screws have been removed. (c) A relief skin incision has been made 6 cm posterior to the skin defect to enable (d) suturing of the defect without causing tension (white arrow). (e) The scalp defect caused by the relief incision portion has been covered with skin graft from the outer layer of skin of the right thigh. (f) Two months after the surgery, the skin incision has healed without infection and the skin graft has been successfully incorporated.

Under general anesthesia, a skin incision was made along the plate to expose it (Figure 2(a)), and then the plate and screws were completely removed (Figure 2(b)). After debridement around the skin defect, a relief skin incision was made 6 cm posterior to it (Figure 2(c)) to enable suturing of the defect portion without causing tension (Figure 2(d), arrow). The relief skin incision portion was covered with a graft from the outer layer of the skin of the right thigh (Figure 2(e)). Two months later, the skin incision had healed without infection and the skin graft had been successfully incorporated (Figure 2(f)).

## 3. Discussion

Exposure of a titanium mini plate several months to several years after cranioplasty or facial bone reconstruction is not rare [6–8]. In our case, exposure of the titanium plate was not caused by infection, which is the most frequent cause of plate protrusion [9]. An edge of the patient's pinwheel-shaped titanium mini plate was bent by relatively minor head trauma, and the upturned part of the plate compressed the scalp, resulting in focal skin ischemia and penetration of the skin. It might be considered that plates utilized for cranioplasty would rarely be bent after being covered by subcutaneous tissue in the absence of infection. However, this case demonstrates that a titanium mini plate can be easily bent by a blow to the scalp directly overlying the plate. Recently, thinner titanium plates (0.3 mm thick) have become more popular for cosmetic reasons [6]. However, the thinner a titanium plate, the easier it would be to bend it. We therefore recommend that screws should be inserted into all holes of pinwheel-shaped titanium mini plates.
